# Longitudinal SARS-CoV-2 Nucleocapsid Antibody Kinetics, Seroreversion, and Implications for Seroepidemiologic Studies

**DOI:** 10.3201/eid2809.220729

**Published:** 2022-09

**Authors:** Michael Loesche, Elizabeth W. Karlson, Opeyemi Talabi, Guohai Zhou, Natalie Boutin, Rachel Atchley, Gideon Loevinsohn, Jun Bai Park Chang, Mohammad A. Hasdianda, Adetoun Okenla, Elizabeth Sampson, Haley Schram, Karen Magsipoc, Kirsten Goodman, Lauren Donahue, Maureen MacGowan, Lewis A. Novack, Petr Jarolim, Lindsey R. Baden, Eric J. Nilles

**Affiliations:** Brigham and Womens Hospital, Boston, Massachusetts, USA (M. Loesche, E.W. Karlson, O. Talabi, G. Zhou, N. Boutin, R. Atchley, G. Loevinsohn, J.B.P. Chang, M.A. Hasdianda, A. Okenla, E. Sampson, H. Schram, K. Magsipoc, K. Goodman, L. Donahue, M. MacGowan, L.A. Novak, P. Jarolim, L.R. Baden, E.J. Nilles);; Massachusetts General Hospital, Boston (M. Loesche, G. Loevinsohn);; Harvard Medical School, Boston (E.W. Karlson, G. Zhou, M.A. Hasdianda, P. Jarolim, L.R. Baden, E.J. Nilles);; Harvard Humanitarian Initiative, Cambridge, Massachusetts, USA (E.J. Nilles)

**Keywords:** SARS-CoV-2, severe acute respiratory syndrome coronavirus 2, coronaviruses, viruses, COVID-19, coronavirus disease, respiratory infections, nucleocapsid antibody, longitudinal, kinetics, seroreversion, seroepidemiology, zoonoses

## Abstract

Given widespread use of spike antibody in generating coronavirus disease vaccines, SARS-CoV-2 nucleocapsid antibodies are increasingly used to indicate previous infection in serologic surveys. However, longitudinal kinetics and seroreversion are poorly defined. We found substantial seroreversion of nucleocapsid total immunoglobulin, underscoring the need to account for seroreversion in seroepidemiologic studies.

Estimating the incidence of infections caused by SARS-CoV-2 that are frequently asymptomatic is challenging when using routine passive surveillance methods. Antibodies can provide a record of previous infection, whether symptomatic or asymptomatic, and serologic surveys that measure antibodies across populations are routinely used for a variety of pathogens and are believed to provide key insights into the epidemiology and transmission of SARS-CoV-2 (*1**–**3*). However, antibody contraction and seroreversion (loss of previously documented antibodies) may lead to false-negative results, a potential issue as we move into the third year of the SARS-CoV-2 pandemic (*4*). Seroepidemiologic studies can adjust for seroreversion when kinetics are well characterized, but limited data are available from >1 year after infection. To characterize seroreversion and ease interpretation of seroepidemiologic studies, we measured SARS-CoV-2 nucleocapsid antibodies, a marker of previous infection even among populations vaccinated with spike-based COVID-19 vaccines, in a longitudinal study of healthcare workers in the United States.

## The Study

The Mass General Brigham Institutional Review Board approved the study protocol (2020P000849). Participants provided written consent to participate in the study.

During April 28‒September 30, 2020, a total of 2,358 employees of the Brigham and Women’s Hospital (Boston, MA, USA) were enrolled in a longitudinal SARS-CoV-2 cohort study. Blood samples were collected at baseline, monthly for 3 months, and then every 3 months through February 2022, up to 21 months after enrollment. Sociodemographic characteristics (Appendix) were collected on electronic questionnaires.

We tested serum samples by using the Diagnostics Elecsys SARS-CoV-2 N Immunoassay (Roche, https://www.roche.com), a double-antigen sandwich electrochemiluminescence total immunoglobulin immunoassay that detects antibodies against viral nucleocapsid protein. We performed assays based on a cutoff index (>1.0) defined according to manufacturer’s guidance. An independent in-house assay performance study that included 832 prepandemic negative controls and 251 PCR-positive samples showed a specificity of 99.6% (95% CI 98.9%–100%) and a sensitivity of 90.8% (95% CI 81.3%–95.7%) for samples collected >14 (range 15–68) days after symptom onset (*5*).

We extracted SARS-CoV-2 PCR test results and dates from the Brigham Health electronic medical record. PCR-positive date was defined as the date of the first registered SARS-CoV-2 PCR-positive test result. For 4 employees who had a positive SARS-CoV-2 PCR test result outside the Brigham Health system, the date of the COVID-19‒positive result, generated for all SARS-CoV-2‒positive employees in the Brigham Health electronic medical record, was used as the PCR-positive date. To assess antibody kinetics, we used a generalized additive mixed-effect model (GAMM) with the natural logarithm of antibody levels modeled as a function of time from first positive PCR test result. To estimate the half-life, we used a linear mixed-effects model (LMM) and assumed constant exponential decay after the peak level (Appendix).

A total of 125 (5.3%) of 2,358 study participants enrolled in the healthcare worker cohort study were positive for nucleocapsid antibodies during April 2020‒January 2021, during predominantly wild-type SARS-CoV-2 circulation (*6*). Of these participants, 110 (88%) had >1 samples collected after an index seropositive sample and were included in the analyses. A total of 687 unique samples were collected from the 110 participants (mean 6.3 samples/participant). The median age of participants was 33 (range 20–71) years; 94 (86%) were female, 97 (88%) White, and 6 (5%) Hispanic. The median body mass index was 24 kg/mm^2^ (range 19–42 kg/mm^2^).

A total of 74 (67%) of 110 participants had a positive SARS-CoV-2 RT-PCR test in the Mass General Brigham electronic health record before the first seropositive sample; 96% were symptomatic (Appendix) and 1 required hospitalization. Symptomatic status was not assessed for study participants who did not have a positive PCR test result because timing of infections was unknown.

**Figure 1:**
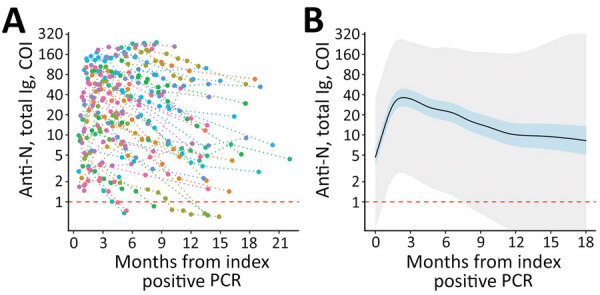
Longitudinal SARS-CoV-2 anti-N total immunoglobulin kinetics among SARS-CoV-2 PCR-positive participants in a study of healthcare workers in Boston, Massachusetts, USA, 2020). A) Individual study participant (n = 74) total anti-N levels from time of index PCR-positive test result. Individual levels are indicated by colored points connected by a dotted line. B) Fitted generalized additive mixed-effect model depicting estimate (solid black line), 95% CI (blue shaded area), and 95% prediction interval (gray shaded area). Estimates are truncated at 18 months given sparsity of later data points. Horizontal dashed red lines indicate the COI for seropositive (above) and seronegative (below) results. The lower limits of detection (COI 0.07) are outside the figure frame. Anti-N, nucleocapsid antibodies; COI, cutoff index.

**Figure 2:**
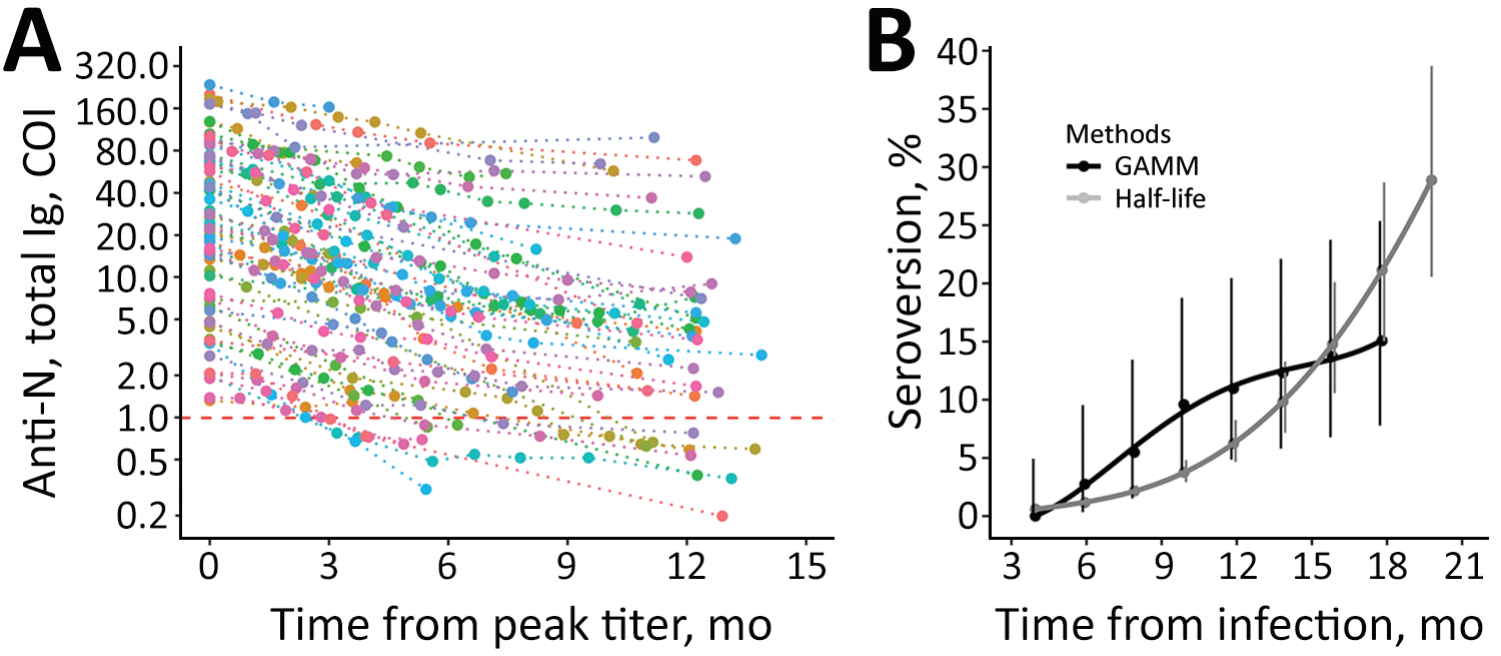
Longitudinal SARS-CoV-2 total anti-N immunoglobulin contraction and estimated seroreversion over time in a study of healthcare workers in Boston, Massachusetts, USA, 2020. A) Consistency in contraction of individual-level total anti-N immunoglobulin levels over time when indexed against participants’ highest recorded sample value. Includes seropositive participants with and without registered SARS-CoV-2 PCR-positive test results and with >1 sampling time points after peak level (n = 90). Individual levels are indicated by colored points connected by a dotted line. Horizontal dashed red line indicates the cutoff index for seropositive (above) and seronegative (below) results. The lower limit of detection (COI 0.07) is outside the figure frame. B) Points indicating estimated rates of seroreversion from total anti-N immunoglobulin seropositive to seronegative by 2-month intervals. The half-life estimate is based on peak total anti-N immunoglobulin level of 37 COI and half-life of 128 days. Solid lines indicate quadratic polynomial trend; error bars indicate 95% CIs. Anti-N, nucleocapsid antibodies; COI, cutoff index; GAMM, generalized additive mixed-effect model.

The mean peak cutoff index level for nucleocapsid antibodies among PCR-positive participants was 37 (95% CI 27–50) and occurred 72 days after the index PCR-positive test result (Figure 1). Assuming constant linear antibody contraction with the LMM approach, antibody half-life was estimated at 128 (95% CI 114–146) days and mean time to seroreversion as 737 (95% CI 680–793) days. Antibody contraction and seroreversion kinetics diverged between the LMM and GAMM models; the nonlinear GAMM model suggested more rapid contraction up to 1 year postinfection, followed by slower contraction and seroreversion thereafter (Table; Figures 1, 2). We observed a stepwise but nonsignificant trend toward a slower relative decrease in concentration of nucleocapsid antibodies among older age groups but not across body mass index or sex (Appendix).

## Conclusions

We report on the kinetics of SARS-CoV-2 nucleocapsid antibodies (total immunoglobulin) up to 21 months after infection and estimate peak antibody levels, kinetics, and rates of seroreversion by using 2 modeling methods. Both models suggest substantial seroreversion by 18 months postinfection, and half-life‒based estimates suggest seroreversion of ≈50% at 2 years.

Half-life‒based approaches that assume constant exponential contraction might underestimate seroreversion through the first year postinfection and overestimate seroreversion at later timepoints (Table). Using a GAMM model that tolerates variable antibody contraction over time, we estimated that seroreversion was 1.4%/month during 4‒12 months after infection, but 95% CIs were wide. Assuming contraction remains relatively constant after an initial rapid decrease, an assumption supported by previous studies on SARS-CoV-2 and other common human coronaviruses (*4*), seroreversion would be 19% at 2 years and 35% at 4 years.

**Table:** SARS-CoV-2 nucleocapsid total immunoglobulin seroreversion by GAMM and half-life methods, by months from infection, among healthcare workers in Boston, Massachusetts, USA, 2020*

Months	GAMM, % (95% CI)	Half-life, % (95% CI)
4	0 (0–4.9)	0.6 (0.6–0.6)
6	2.7 (3.0–9.5)	1.2 (1.0–1.3)
8	5.5 (1.5–13.4)	2.1 (1.8–2.6)
10	9.6 (3.9–18.8)	3.8 (2.9- 4.8)
12	11 (4.9–20.5)	6.2 (4.7–8.3)
14	12.3 (5.8–22.1)	9.8 (7.2–13.3)
16	13.7 (6.8–23.8)	14.8 (10.6–20.1)
18	15.1 (7.8–25.4)	21.1 (15.0–28.7)
24	NC	48.9 (36.1–62.1)

*The GAMM method estimates are restricted to observational data and therefore not calculated more than 18 months postinfection. Half-life method represents constant log linear antibody contraction with an estimated peak total nucleocapsid antibody level that had a cutoff index of 37 and a half-life of 128 days. GAMM, generalized additive mixed model; NC, not calculated.

These findings suggested that serologic surveys conducted >1 year after widespread SARS-CoV-2 transmission will be markedly affected by seroreversion. The total immunoglobulin immunoassay used for this study shows more durable detection of nucleocapsid antibodies than other formats, particularly single isotope assays, and rates of seroreversion might be higher across other assay designs (*5*,*7*). Total immunoglobulin immunoassays specific for spike antibodies versus nucleocapsid antibodies appear to provide improved durability of antibody detection, and single isotype antispike assays provide similar or lower durability than total nucleocapsid immunoglobulin assays (*7*). However, given the widespread use of spike-based COVID-19 vaccines, the utility of spike antibodies for detection of previous infection in population-level surveys is limited.

This study has several strengths, including a mean of 6.3 unique sample time points/participant that enables more precise demarcation of antibody dynamics and a long study interval that includes samples collected up to 21 months postinfection. However, our cohort was based in the United Sates, enrolled only adults, and overrepresented women and White participants. Therefore, our findings might not be generalizable. Peak antibody levels were estimated for PCR-positive persons; lower peak levels might be observed among seropositive persons who do not provide a PCR-positive test result, which would cause more rapid seroreversion than that we report. Data points >18 months postinfection are sparse, and antibody dynamics might differ outside our measurement window. Infections accrued during wild-type predominance, in the prevaccination setting, are assumed to reflect a single infection. Antibody kinetics might differ for infections caused by other SARS-CoV-2 strains or after vaccine breakthrough or repeat infections (H.J. Whitaker et al., unpub. data, https://www.medrxiv.org/content/10.1101/2021.10.25.21264964v1.full-text; D. Follman et al., unpub. data, https://www.medrxiv.org/content/10.1101/2022.04.18.22271936v1.full).

In summary, antibody seroreversion and the global rollout of vaccines are increasingly useful considerations when planning and interpreting SARS-CoV-2 seroepidemiologic studies. Immunoassays targeting the nucleocapsid protein can detect previous infection among populations vaccinated with spike protein vaccines, but antibody contraction and seroreversion are likely to be substantial as we move into the third year of the pandemic. By characterizing seroreversion after SARS-CoV-2 infection, this study provides provisional format-specific considerations for interpreting and adjusting estimates of previous infection for seroepidemiologic purposes.

Appendix Additional information on longitudinal SARS-CoV-2 nucleocapsid antibody kinetics, seroreversion, and implications for seroepidemiologic studies.

## References

[1] Kleynhans J, Tempia S, Wolter N, von Gottberg A, Bhiman JN, Buys A, et al.; PHIRST-C Group. PHIRST-C Group. SARS-CoV-2 seroprevalence in a rural and urban household cohort during first and second waves of infections, South Africa, July 2020‒March 2021. Emerg Infect Dis. 2021;27:3020–9. 10.3201/eid2712.211465 34477548 PMC8632160

[2] O’Driscoll M, Ribeiro Dos Santos G, Wang L, Cummings DAT, Azman AS, Paireau J, et al. Age-specific mortality and immunity patterns of SARS-CoV-2. Nature. 2021;590:140–5. 10.1038/s41586-020-2918-0 33137809

[3] Clarke KEN, Jones JM, Deng Y, Nycz E, Lee A, Iachan R, et al. Seroprevalence of Infection-Induced SARS-CoV-2 Antibodies - United States, September 2021-February 2022. MMWR Morb Mortal Wkly Rep. 2022;71:606–8. 10.15585/mmwr.mm7117e3 35482574 PMC9098232

[4] Harris RJ, Whitaker HJ, Andrews NJ, Aiano F, Amin-Chowdhury Z, Flood J, et al. Serological surveillance of SARS-CoV-2: Six-month trends and antibody response in a cohort of public health workers. J Infect. 2021;82:162–9. 10.1016/j.jinf.2021.03.015 33766553 PMC7982645

[5] Nilles EJ, Karlson EW, Norman M, Gilboa T, Fischinger S, Atyeo C, et al. Evaluation of three commercial and two non-commercial immunoassays for the detection of prior infection to SARS-CoV-2. J Appl Lab Med. 2021;6:1561–70. 10.1093/jalm/jfab072 34196711 PMC8420636

[6] Brown C, Doucette M, Fink T, Gallagher G, Lang A, Madoff L, et al. Detection of a large cluster and multiple introductions of the P.1 SARS-CoV-2 variant of concern in Massachusetts: SARS-CoV-2 coronavirus/nCoV-2019 genomic epidemiology, virological, 2021 [cited 2022 Jun 9]. https://virological.org/t/detection-of-a-large-cluster-and-multiple-introductions-of-the-p-1-sars-cov-2-variant-of-concern-in-massachusetts/671

[7] Stone M, Grebe E, Sulaeman H, Di Germanio C, Dave H, Kelly K, et al. Evaluation of commercially available high-troughput SARS-CoV-2 serologic assays for serosurveillance and related applications. Emerg Infect Dis. 2022;28:672–83. 10.3201/eid2803.211885 35202525 PMC8888213

