# Supplementary material for: Longitudinal SARS-CoV-2 Nucleocapsid Antibody Kinetics, Seroreversion, and Implications for Seroepidemiologic Studies
Source: Emerg Infect Dis. 2022 Sep;28(9):1859–62. doi: 10.3201/eid2809.220729 (PMC9423917; doi:10.3201/eid2809.220729)
Supplement: Appendix — Additional information on longitudinal SARS-CoV-2 nucleocapsid antibody kinetics, seroreversion, and implications for seroepidemiologic studies. [file 22-0729-Techapp-s1.pdf]

Article DOI: https://doi.org/10.3201/eid2809.220729

Longitudinal SARS-CoV-2 Nucleocapsid Antibody Kinetics, Seroreversion, and Implications for Seroepidemiologic Studies

Appendix

Methods

Generalized Additive Mixed-Effect Model

A generalized additive mixed-effect model (GAMM) was generated to model SARS-CoV-2 antibody titers over time. The natural logarithm of antibody titers was modeled as a function of time from first positive SARS-CoV-2 PCR test result, which was incorporated as a nonparametric smoothing function with a cubic regression basis function. A random intercept and smoothing function was used with subjects as the grouping variable. The default value for knots was used. The model was fit by maximum likelihood with a Gaussian link function. The fitted GAMM was used to estimate peak antibody titers and time until peak. Final model specifics and measures of model fit are provided.

Linear Mixed-Effects Model

To estimate the rate of decay of antibody levels, we assumed that there was a constant exponential decay following peak titer. A linear mixed-effects model (LMM) was fitted on antibody titer levels centered on the peak measured antibody date. Random intercept and slope were use with subjects as the grouping variable. This model was used to calculate the half-life interval. The LMM was fitted by restricted maximum likelihood with a Gaussian link function. Estimated seroreversion is computed using the GAM fit which incorporated the nonlinear pattern of slower waning 12 months post infection plus a LMM fit (which estimates individual deviations from the GAMM).

Statistical analyses were performed by using the R statistical programming language (R Core Team 2021, https://www.r-project.org). The mgcv (https://cran.r-project.org/web/packages/mgcv/index.html) and LME4 (https://cran.r-

project.org/web/packages/lme4/index.html) packages were used for the GAMM and LMM models respectively.

Appendix Table 1. Variables for study participants (n = 110)

Variable	No. participants (%)
Age, y	
20–29	43 (39)
30–49	45 (41)
≥50	22 (20)
Sex	
F	94 (85)
M	15 (14)
Not reported	1 (1)
Race	
Asian	3 (3)
Black	7 (6)
White	97 (88)
>1	1 (1)
Not reported	2 (2)
Ethnicity	
Hispanic	6 (5)
Non-Hispanic	100 (91)
Not reported	4 (4)
Body mass index, kg/mm^2	
18–24	65 (59)
25–29	27 (25)
≥30	13 (12)
Not reported	5 (5)
Concurrent conditions	
Diabetes	0 (0)
Asthma	11 (10)
High blood pressure	10 (9)
Coronary heart disease	1 (1)
Cerebral vascular accident (stroke)	0 (0)
Immunocompromised (HIV)	1 (1)

Appendix Table 2. Clinical features reported by SARS-CoV-2 PCR positive study participants (n = 70)*

Reported clinical feature	No. (%)
Measured fever (temperature ≥38.0°C)	9 (13)
Subjective fevers or chills	30 (43)
Cough	41 (59)
Shortness of breath	16 (23)
Sore throat	25 (36)
Nasal congestion or rhinorrhea	40 (57)
Nausea, vomiting, or diarrhea	12 (17)
Ageusia or anosmia	24 (34)
Chest pain or pressure	14 (20)
Myalgias or arthralgias	33 (47)
Fatigue	22 (31)
Any symptom	67 (96)

*Data were extracted from the Brigham Health electronic medical records system by using a standardized data extraction form. Symptoms reported within 14 d before or after the index PCR-positive date were included. Of 74 total participants with a registered PCR-positive SARS-CoV-2 test result, sufficient detail was available to assess symptoms for 70. Given timing of SARS-CoV-2 infection is not available for seropositive participants that did not register a positive PCR-test result, these study participants (n = 36) were excluded from assessment of clinical features.

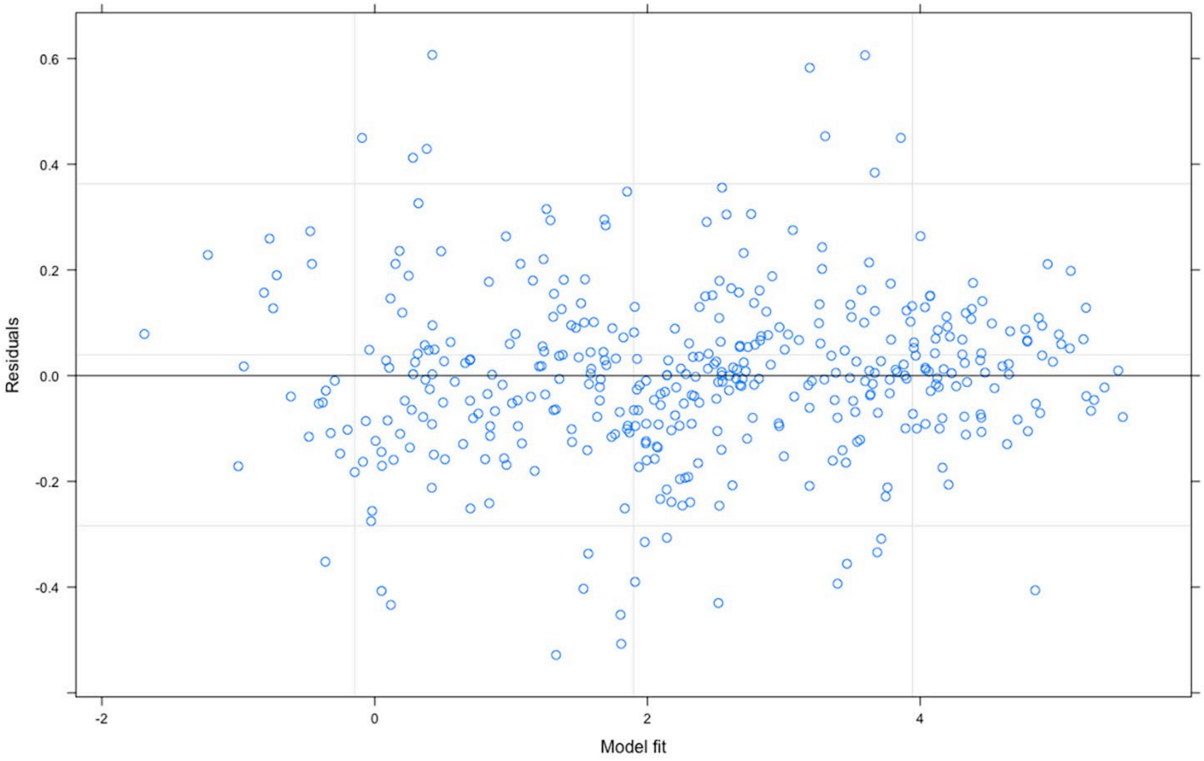

Appendix Figure 1. Residuals plot of the linear mixed effect model used to estimate rate of nucleocapsid antibody contraction. Individual variation in rate of decay accounted for 29.6% of the variance.

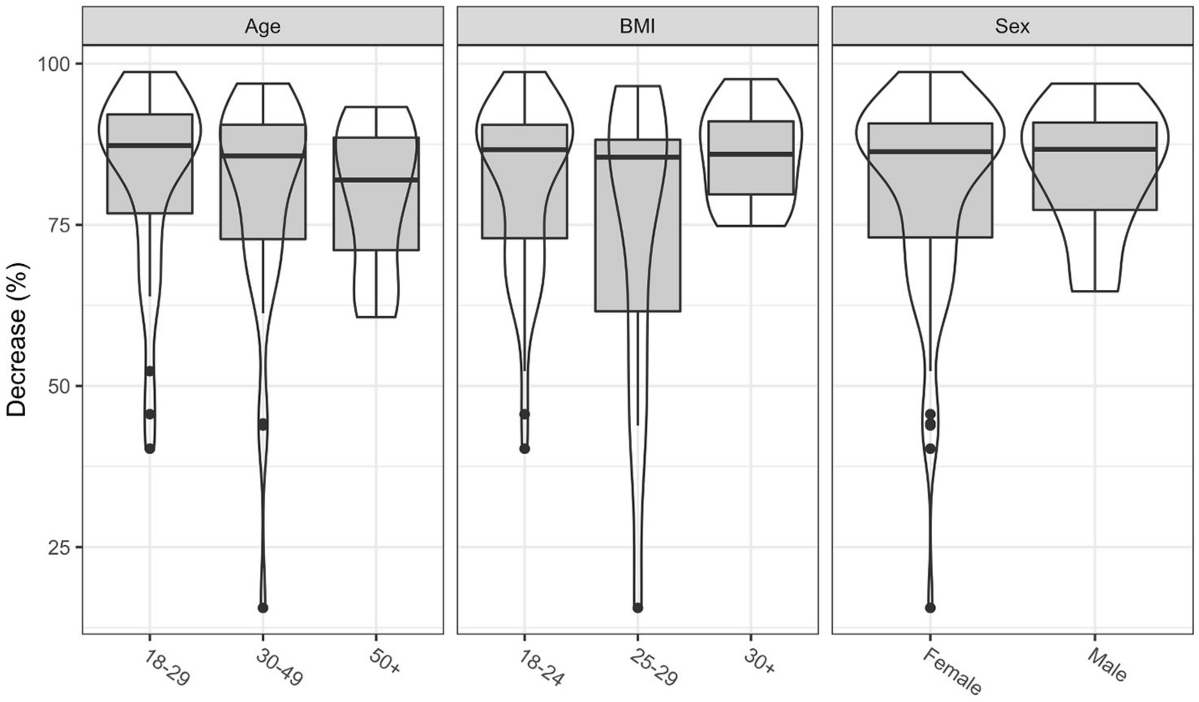

Appendix Figure 2. Box plots depicting the distribution of individual level rate of decay for subjects reported as percent decrease from peak at 1 year stratified by demographic features. Horizonal black bar indicates median value, box plot limits indicate Q1–Q3, vertical line indicates minimum and maximum values without outliers, and dots indicate lower (Q1–1.5 IQR) and upper (Q3 + 1.5 IQR) outliers. Overlaid violin plots show distribution. Given the limited number of participants, statistical differences are not identified, but stepwise declines in percent decrease per year with increasing age suggest potential differences in durability of nucleocapsid antibodies by age might exist. BMI, body mass index; IQR, interquartile range.
